# Structural analysis of retinal blood vessels in patients with COPD during a pulmonary rehabilitation program

**DOI:** 10.1038/s41598-019-56997-5

**Published:** 2020-01-08

**Authors:** Anouk W. Vaes, Martijn A. Spruit, Karel Van Keer, João Barbosa-Breda, Emiel F. M. Wouters, Frits M. E. Franssen, Jan Theunis, Patrick De Boever

**Affiliations:** 1 Research and Education, Ciro, Horn, Netherlands; 2 Health Unit, Flemish Institute for Technological Research (VITO), Mol, Belgium; 3 Department of Respiratory Medicine, Maastricht University Medical Centre (MUMC+), Maastricht, The Netherlands; 4 NUTRIM School of Nutrition and Translational Research in Metabolism, Maastricht, The Netherlands; 5 REVAL - Rehabilitation Research Center, BIOMED - Biomedical Research Institute, Faculty of Rehabilitation Sciences, Hasselt University, Diepenbeek, Belgium; 6 Research Group Ophthalmology, Department of Neurosciences, KU Leuven, Leuven, Belgium; 7 Ophthalmology Department, Centro Hospitalar Sao Joao, Porto, Portugal; 8 Department of Surgery and Physiology, Faculty of Medicine of the University of Porto, Porto, Portugal; 9 Centre for Environmental Sciences, Hasselt University, Hasselt, Belgium

**Keywords:** Chronic obstructive pulmonary disease, Cardiovascular diseases

## Abstract

Cardiovascular diseases are frequently present in chronic obstructive pulmonary disease (COPD). Population-based studies found associations between retinal vessel diameters and cardiovascular health, but it is unknown whether this also applies to COPD patients. Therefore, we measured retinal vessel diameters in COPD patients and aimed to determine the association with cardiovascular risk factors, lung function, and functional outcomes. In addition, we investigated whether an exercise-based pulmonary rehabilitation (PR) program would change retinal vessel diameters, as a proxy for improved microvascular health. Demographics and clinical characteristics, including pulmonary function, exercise capacity, blood pressure, blood measurements and level of systemic inflammation were obtained from 246 patients during routine assessment before and after PR. Retinal vessel diameters were measured from digital retinal images. Older age and higher systolic blood pressure were associated with narrower retinal arterioles (β: −0.224; p = 0.042 and β: −0.136; p < 0.001, respectively). Older age, higher systolic blood pressure and lower level of systemic inflammation were associated with narrower retinal venules (β: −0.654; −0.229; and −13.767, respectively; p < 0.05). No associations were found between retinal vessel diameters and lung function parameters or functional outcomes. After PR, no significant changes in retinal venular or arteriolar diameter were found. To conclude, retinal vessel diameters of COPD patients were significantly associated with systolic blood pressure and systemic inflammation, whilst there was no evidence for an association with lung function parameters, functional outcomes or other cardiovascular risk factors. Furthermore, an exercise-based PR program did not affect retinal vessel diameter.

## Introduction

Chronic obstructive pulmonary disease (COPD) is characterized by persistent respiratory symptoms and airflow limitation due to airway and/or alveolar abnormalities usually caused by significant exposure to noxious particles or gases^1^. Although the diagnosis of COPD is based on spirometry, it is well recognized that COPD is associated with a range of systemic extrapulmonary effects^1^. Indeed, comorbidities are frequently present and contribute to the severity of disease^2–4^. Cardiovascular disease (including hypertension, coronary artery disease, congestive heart failure, stroke and peripheral arterial disease) is a common comorbidity in COPD^2,5^.

Smoking is the most important risk factor for the development of COPD, but it is also a major risk factor for the development of cardiovascular disease^6,7^. Indeed, cigarette smoke not only affects the airway epithelium, but also causes vascular endothelial damage and loss of microvascular integrity in several organs, including the heart, brain, and kidney^8,9^. In addition, systemic inflammatory processes, oxidative stress, and hypoxia may contribute to microvascular complications in COPD^10,11^. Since it has been suggested that changes in the microvasculature may be associated with cardiovascular disease^12,13^, monitoring the properties of the microvascular system in COPD could be valuable for additional phenotyping with possible added-value in clinical practice for cardiovascular risk stratification^14^.

Retinal microvasculature can be easily visualized using fundus photography and can serve as a non-invasive proxy to assess the microvascular health status^15^. Findings from large epidemiological studies demonstrated that structural assessment of retinal microvasculature can provide useful information for cardiovascular risk prediction^16–19^. Indeed, it has been recognized that changes in the retinal microvasculature may precede the occurrence of hypertension, obesity and diabetes mellitus (DM)^20^. Earlier population-based studies demonstrated that more narrow retinal arteriolar vessels are associated with hypertension, while wider retinal venular vessels are associated with systemic inflammatory markers, hyperglycaemia, obesity and dyslipidaemia^18,21^. Similar findings were observed in patients with type 2 DM, heart failure or rheumatoid arthritis^22–25^.

To date, systematic studies characterizing the retinal microvasculature in patients with COPD are scarce. Data from a population study suggest that the width of retinal venules is inversely associated with forced expiratory volume in one second (FEV_1_) and FEV_1_/forced vital capacity (FVC)^26,27^. This would indicate that vascular comorbidities associated with obstructive decrements in lung function may be characterized by defects in the microvascular circulation, as assessed with fundus imaging^26^.

The primary aim of this study was to measure retinal vessel widths in patients with COPD and to determine the association between these metrics and lung function parameters, functional outcomes and cardiovascular risk factors. *A priori*, we expected an inverse association between retinal vessel diameter and lung function parameters in patients with COPD^26,27^. Moreover, based on similarities with type 2 DM and heart failure, we postulated that the microcirculation, as assessed by retinal vessel diameters, is inversely associated with systemic cardiometabolic parameters and functional outcomes in patients with COPD^22–24^.

In addition, we aimed to determine the effects of an exercise-based pulmonary rehabilitation program on retinal vessel diameters in patients with COPD. Based on earlier work, demonstrating that regular exercise can induce retinal arteriolar dilatation and venular constriction in healthy adults^28,29^, we hypothesized that regular exercise training can change the dimensions of retinal vessel diameters in these patients and hence provide support for improved microcirculatory status as a result of regular exercise.

## Results

**Figure 1:**
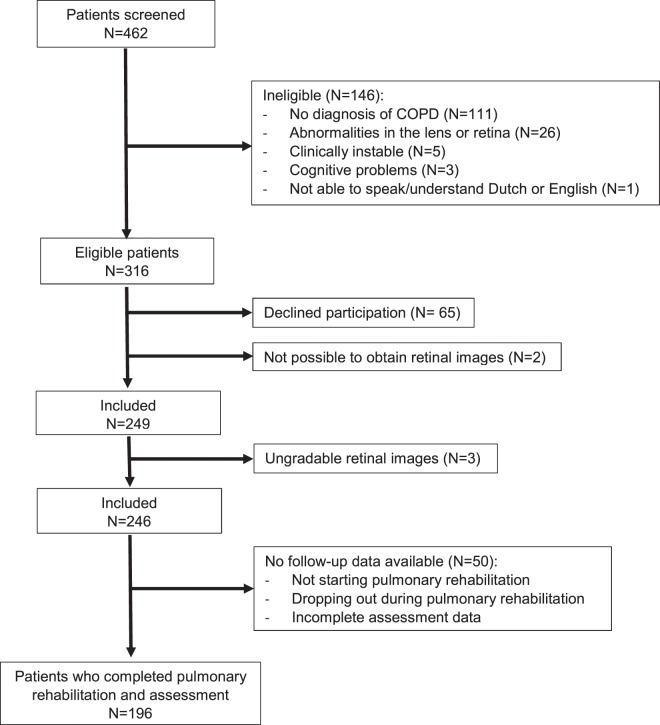
Study flow chart: screening and enrollment of study participants.

Four hundred sixty-two patients with COPD referred for pulmonary rehabilitation at Ciro were screened between July 2016 and June 2017. One hundred forty-six patients were ineligible to participate due to various reasons (Fig. 1). In addition, 65 eligible subjects declined participation because of disinterest, and in two patients it was not possible to obtain retinal images due to small pupils. Two hundred forty-nine patients underwent fundus photography, of which three patients had ungradable retinal images. So finally, 246 patients were included in the analyses.

### Characteristics

Patient characteristics are listed in Table 1. Slightly more than half of the patients were male and mean age was 64.4 ± 8.5 years. Patients had mild to very severe COPD and were frequently diagnosed with risk factors of cardiovascular diseases, including hypertension, (pre-) DM, and dyslipidemia. Furthermore, 47.6% of the patients had a history of cardiovascular diseases, including coronary artery disease (20.8%), peripheral arterial disease (10.4%), stroke (8.0%) and congestive heart failure (6.8%). Patients showed impaired aerobic capacity, as assessed by cardiopulmonary exercise test (CPET) and six-minute walk test (6MWT). Long-term oxygen therapy was used by 18.3% of the patients.

**Table 1:** Baseline characteristics of COPD patients (n = 246).

Clinical characteristics			
Sex, male, %			56.1
Age, years			64.4 (8.5)
Body mass index, kg/m^2^			26.2 (5.8)
Fat free mass index, kg/m^2^			16.3 (2.5)
Smoking (%)		Current	28.0
		Ex	65.9
		Never	6.1
Smoking pack-years			41.6 (24.7)
FEV_1_, L			1.27 (0.64)
FEV_1_, % pred			47.3 (19.9)
FEV_1_/FVC			35.9 (13.0)
TL_CO_, % pred			51.1 (16.7)
RV, % pred			164.9 (53.7)
TLC, % pred			118.8 (18.6)
GOLD I/II/III/IV (%)			7.7/28.0/46.7/17.5
GOLD A/B/C/D (%)			1.6/12.6/7.3/78.5
Long-term oxygen therapy (%)			18.3
**Blood gases**			
O_2_ saturation at rest, %			93.7 (2.8)
PaO_2_ kPa			9.3 (1.4)
PaCO_2_ kPa			5.5 (0.9)
**Blood pressure**			
Systolic blood pressure, mmHg			130.9 (23.7)
Diastolic blood pressure, mmHg			77.2 (11.3)
Hypertension, %			68.3
**Laboratory values**			
Blood glucose, mmol/L			5.7 (1.1)
(Pre) diabetes mellitus, %			24.0
Dyslipidemia, %			58.1
hs-CRP	low (%)		24.6
	borderline (%)		31.7
	moderately high (%)		29.6
	markedly high (%)		14.2
**COPD medication, %**			
SABA			54.4
LABA			14.8
SAMA			9.6
LAMA			61.2
ICS			18.4
ICS/LABA combination			55.6
LAMA/LABA combination			20.8
SAMA/SABA combination			19.6
**Cardiovascular medication, %**			
Statins			39.8
Antihypertensives*			45.1
Anticoagulants			10.2
Antiarrythmics			8.5
**Cardiovascular comorbidities, %**			
Coronary artery disease			20.8
Peripheral arterial disease			10.4
Stroke			8.0
Congestive heart failure			6.8
**Exercise capacity**			
Peak VO_2_, mL/min			1096 (352)
Peak VO_2_, %predicted			60.2 (17.8)
6-minute walk distance, m			400 (109)
6-minute walk distance, %predicted			62.8 (16.8)
Constant work rate test, s			312 (229)
**Retinal vessel diameter**			
CRAE, µm			152.5 (12.7)
CRVE, µm			233.7 (21.1)

kg = kilogram; m = meter; mMRC = modified Medical Research Council; FEV_1_ = forced expiratory volume in the first second; FVC = forced vital capacity; O_2_ = oxygen; PaO_2_ = arterial partial pressure of oxygen; kPa = kilopascal; PaCO_2_ = arterial partial pressure of carbon dioxide; mm Hg = millimeter of mercury; L = liter; hs-CRP = high-sensitive C-reactive protein; SABA = short-acting beta-agonist; LABA = long-acting beta-agonist; SAMA = short-acting muscarinic antagonist; LAMA = long-acting muscarinic antagonist; ICS = inhaled corticosteroids; VO_2_ = oxygen uptake; min = minute; CRAE = central retinal arteriolar equivalent; CRVE = central retinal venular equivalent *i.e. ACE inhibitors, beta-blockers, diuretics, calcium channel blockers, angiotensin II receptor antagonists.

### Retinal findings

Patients had a mean central retinal arteriolar equivalent (CRAE) of 152.5 ± 12.7 µm and mean central retinal venular equivalent (CRVE) of 233.7 ± 21.1 µm. Retinal abnormalities were present in 146 (59%) patients, of which 47 (19%) had multiple abnormalities. Most common abnormalities were increased vessel tortuosity (n = 44), peri-papillary atrophy (n = 42), (suspicion of) cataract (n = 34), and (suspicion of) glaucoma (n = 22) (See Supplementary Table S1).

### Associations with retinal vessel diameters

Gender and age-adjusted univariate associations between retinal vessel diameter and lung function parameters, clinical variables, cardiovascular risk factors and functional outcomes of pulmonary rehabilitation are listed in Table 2. Older patients with COPD had a significantly smaller CRAE and CRVE. Several other variables were inversely associated with CRAE, including transfer factor for carbon monoxide (TL_CO_ %predicted), arterial oxygen saturation at rest (SaO_2_), systolic blood pressure, diastolic blood pressure, and triglycerides levels (Table 2). In addition, SaO_2_ and systolic blood pressure were inversely associated with CRVE, whilst low-density lipoprotein cholesterol levels and level of systemic inflammation were positively associated with CRVE (Table 2). Results did not demonstrate an association between retinal vessel diameters and FEV_1_ or FEV_1_/FVC. Furthermore, no association was found between retinal vessel widths and baseline exercise capacity (Table 2).

**Table 2:** Age- and sex-adjusted association between CRAE and CRVE and clinical, cardiovascular and functional measures.

				CRAE		CRVE	
				Β	p-value	Β	p-value
**Clinical characteristics**							
Sex, male*				−0.424	0.795	3.472	0.198
Age, years*				−0.351	<0.001	−0.621	0.000
Body mass index, kg/m^2^				−0.187	0.170	0.087	0.698
Fat free mass index, kg/m^2^				−0.171	0.634	0.308	0.601
Smoking (%)		Current		1.965	0.578	−10.522	0.068
		Ex		−0.124	0.971	−6.760	0.220
		Never		1		1	
Smoking pack-years				−0.004	0.907	−0.007	0.898
FEV_1_, % pred				0.012	0.753	−0.008	0.905
FEV_1_/FVC				−0.009	0.888	0.008	0.937
TL_CO_, % pred				−0.118	0.016	−0.060	0.456
RV, % pred				−0.002	0.882	−0.021	0.413
TLC, % pred				0.005	0.912	−0.046	0.542
GOLD stage			I	−3.231	0.338	−1.564	0.779
			II	2.946	0.218	4.274	0.280
			III	−0.850	0.701	2.288	0.533
			IV	1		1	
Long-term oxygen therapy				4.567	0.310	3.173	0.462
**Blood gases**							
O_2_ saturation at rest, %				−0.576	0.037	−0.934	0.039
PaO_2_, kPa				−0.898	0.102	−1.492	0.097
PaCO_2_, kPa				0.507	0.565	1.100	0.445
**Blood pressure**							
Systolic blood pressure, mmHg				−0.102	0.003	−0.137	0.016
Diastolic blood pressure, mmHg				−0.163	0.020	−0.030	0.795
Hypertension, %				2.313	0.207	0.140	0.963
**Laboratory values**							
Blood glucose, mmol/L				−1.290	0.073	−2.294	0.052
(Pre) diabetes mellitus, %				−0.434	0.807	2.222	0.444
Total cholesterol				−0.271	0.967	1.227	0.281
HDL				−0.289	0.836	−2.404	0.290
LDL				0.182	0.811	2.513	0.041
Triglycerides				−2.120	0.043	−1.914	0.267
Dyslipidemia, %				0.234	0.885	−0.815	0.759
hs-CRP	low (%)			4.002	0.130	−12.857	0.050
	borderline (%)			0.389	0.877	−5.412	0.028
	moderately high (%)			−1.042	0.683	−0.624	0.883
	markedly high (%)			1		1	
**COPD medication, %**							
Beta-agonists				−2.016	0.449	−5.872	0.214
Anticholinergics				−2.095	0.501	−2.267	0.657
Inhaled corticosteroids				1.763	0.383	0.799	0.810
ICS/LABA combination				1.289	0.420	3.197	0.222
LAMA/LABA combination				1.460	0.451	−0.734	0.817
SAMA/SABA combination				−2.122	0.350	−1.698	0.600
**Cardiovascular medication, %**				−1.133	0.530	−0.018	0.995
Statins				0.883	0.603	−0.065	0.981
Antihypertensives**				−1.431	0.387	−0.005	0.999
Anticoagulants				1.764	0.301	−0.529	0.850
Antiarrythmics				0.615	0.815	−1.196	0.781
**Cardiovascular comorbidities, %**				0.965	0.566	−1.637	0.557
**Exercise capacity**							
Peak VO_2_, mL/min†				−0.003	0.363	0.003	0.492
6-minute walk distance, m				−0.008	0.278	0.005	0.716
Constant work rate test, s‡				0.004	0.293	0.008	0.298

^*^Age and sex were modeled separately, not adjusting for the other variable.

^†^available in 196 patients; ‡available in 223 patients.

kg = kilogram; m = meter; FEV_1_ = forced expiratory volume in the first second; FVC = forced vital capacity; O_2_ = oxygen; PaO_2_ = arterial partial pressure of oxygen; kPa = kilopascal; PaCO_2_ = arterial partial pressure of carbon dioxide; mm Hg = millimeter of mercury; L = liter; hs-CRP = high-sensitive C-reactive protein; SABA = short-acting beta-agonist; LABA = long-acting beta-agonist; SAMA = short-acting muscarinic antagonist; LAMA = long-acting muscarinic antagonist; ICS = inhaled corticosteroids; VO_2_ = oxygen uptake; min = minute.

**i.e. ACE inhibitors, beta-blockers, diuretics, calcium channel blockers, angiotensin II receptor antagonists

Multivariate regression models for CRAE and CRVE are listed in Table 3 (See Supplementary Table S2 for the complete model with all covariates). Model 1 identifies an association between older age and higher systolic blood pressure and narrower retinal arterioles (β: −0.224 (95% confidence interval (CI) −0.439, −0.009), p = 0.042; and β: −0.136 (95%CI −0.210, −0.062), p < 0.001) when adjusting for relevant confounders, including sex, body mass index (BMI), fat free mass index (FFMI), FEV_1_/FVC, TL_CO_, residual volume (RV), total lung capacity (TLC), diastolic blood pressure, arterial partial pressure of oxygen (PaO_2_), long-term oxygen therapy, smoking status, smoking pack-years, blood glucose, dyslipidemia, pulmonary and cardiovascular medication, cardiovascular comorbidities and fellow vessel diameter. Higher systolic blood pressure, low level of systemic inflammation and older age were associated with narrower retinal venules; β: −0.229 (95%CI −0.379, −0.120), p = 0.041; −13.767 (95%CI −24.556, −2.978), p < 0.001; and −0.654 (95%CI −1.089, −0.381), p = 0.042; respectively. These associations remained significant after further adjustment for fellow retinal vessel diameter (model 2; Table 3).

**Table 3:** Multiple regression model of CRAE and CRVE.

		CRAE (µm) Model 1		CRAE (µm) Model 2		CRVE (µm) Model 1		CRVE (µm) Model 2	
		β (95% confidence interval)	P-value	β (95% confidence interval)	P-value	β (95% confidence interval)	P-value	β (95% confidence interval)	P-value
Age, years		−0.224 (−0.439 - −0.009)	0.042	−0.193 (−0.402 - −0.016)	0.047	−0.654 (−1.089 - −0.381)	0.042	−0.442 (−0.868 - −0.083)	0.044
Systolic blood pressure, mmHG		−0.136 (−0.210 - −0.062)	<0.001	−0.120 (−0.193 - −0.048)	<0.001	−0.229 (−0.379 - −0.120)	0.041	−0.211 (−0.370 - −0.047)	0.047
hs-CRP	low (%)	—	—	—	—	−13.767 (−24.556 - −2.978)	<0.001	−15.493 (−25.987 - −4.998)	<0.001
	moderate (%)	—	—	—	—	−15.097 (−25.521 - −4.674)	<0.001	−16.496 (−26.624 - −6.368)	<0.001
	borderline (%)	—	—	—	—	−2.309 (−4.833 - −0.785)	0.022	−3.768 (−6.995 - −0.541)	0.008
	high (%)	—	—	—	—	0	.	0	.

Model 1 adjusted for sex, age and relevant confounders, including BMI, FFMi, FEV_1_/FVC, TL_CO_, RV, TLC, diastolic blood pressure, PaO_2_, long-term oxygen therapy, smoking status, smoking pack-years, blood glucose, total cholesterol, HDL cholesterol, LDL cholesterol, triglycerides, cardiovascular medication; Model 2 adjusted for all variables in model 1 plus fellow vessel diameter (i.e. CRVE in model for CRAE or vice versa).

The complete models are included in the online supplement (Supplementary Table S2 and S3).

### Response to pulmonary rehabilitation

One hundred and ninety-six patients with COPD completed pulmonary rehabilitation and assessment after rehabilitation. Characteristics of these patients are listed in Table 4. Baseline characteristics of these patients were not significantly different compared to patients who did not complete pulmonary rehabilitation and assessment after rehabilitation.

**Table 4:** Baseline characteristics of COPD patients stratified according to response to pulmonary rehabilitation.

	All COPD patients (n = 196)	Non-responder (n = 67)	≥100 s change on CWRT or ≥30 m change on 6MWT (n = 90)	≥100 s change on CWRT + ≥30 m change on 6MWT (n = 39)
**Clinical characteristics**				
Sex, male, %	57.7	52.2	54.4	64.4
Age, years	63.7 (8.3)	64.8 (8.0)	63.3 (8.8)	63.0 (7.7)
Body mass index, kg/m^2^	26.2 (5.7)	24.7 (4.6)	26.4 (5.3)	28.3 (7.6)*
Fat free mass index, kg/m^2^	16.4 (2.5)	16.0 (2.2)	16.4 (2.6)	17.5 (2.7)*
FEV_1_, L	1.30 (0.63)	1.18 (0.56)	1.32 (0.67)	1.45 (0.63)*
FEV_1_, % pred	48.1 (19.5)	46.4 (20.2)	48.7 (19.9)	49.6 (17.4)
FEV_1_/FVC	36.1 (13.0)	33.1 (11.1)	36.7 (13.6)	39.7 (13.7)*
**Exercise capacity**				
Peak VO_2_, mL/min	1106 (343)	1080 (296)	1097 (365)	1177 (370)
Peak VO_2_, %predicted	60.7 (17.5)	64.0 (17.0)	59.6 (18.3)	57.5 (16.0)
6-minute walk distance, m	419 (97)	427 (91)	423 (100)	394 (97)
6-minute walk distance, %predicted	65.4 (15.1)	67.1 (15.0)	66.2 (15.2)	60.8 (14.2)
Change in 6-minute walk distance, m	14 (50)	−21 (44)	16 (39)*	69 (30)*†
Constant work rate test, s	312 (230)	333 (309)	301 (185)	301 (153)
Change in constant work rate test, s	224 (358)	−53 (205)	321 (340)*	473 (301)*†
**Retinal findings**				
CRAE, µm	152.9 (12.3)	152.1 (13.1)	153.4 (12.0)	152.8 (11.5)
Change in CRAE, µm	0.02 (7.2)	1.84 (6.0)	−1.21 (6.3)	−0.37 (10.2)
CRVE, µm	234.8 (19.9)	234.1 (23.6)	235.9 (18.9)	233.6 (15.3)
Change in CRVE, µm	0.61 (11.1)	0.89 (12.4)	−0.40 (10.0)	2.41 (10.9)
Retinal abnormalities, %	59	66	56	59

kg = kilogram; m = meter; mMRC = modified Medical Research Council; FEV_1 _= forced expiratory volume in the first second; FVC = forced vital capacity; L = liter; VO_2_=oxygen uptake; min = minute; s = seconds; CRAE = central retinal arteriolar equivalent; CRVE = central retinal venular equivalent.

*p < 0.05 vs. non-responder; ^†^p < 0.05 vs. ≥100 s change on CWRT or ≥30 m change on 6MWT.

After pulmonary rehabilitation, patients had a significantly improved six-minute walk distance (14 ± 50 m, p = 0.001) and cycle endurance time on constant work rate test (CWRT) (224 ± 358 s, p < 0.001). 33.5% of the patients achieved a clinically relevant improvement in 6MWT, whilst 51.5% of the patients achieved a clinically relevant improvement in cycle endurance time. No significant differences were found in CRAE (0.02 ± 7.2 µm, p = 0.97) and CRVE (0.61 ± 11.1 µm, p = 0.52) following pulmonary rehabilitation.

Non-responders to pulmonary rehabilitation had a significantly worse baseline lung function, and lower BMI and FFMI compared to responders. Baseline retinal vessel diameters and changes in retinal vessel diameters after pulmonary rehabilitation were not significantly different between responders and non-responders (Table 4). In addition, baseline CRAE or CRVE were not significantly associated with change in functional outcomes of pulmonary rehabilitation, including six-minute walk distance (β for CRAE: −0.002 (95%CI −0.035, 0.032); p = 0.930 and β for CRVE: −0.007 (95%CI −0.062, 0.047); p = 0.792, respectively) and cycle endurance time on CWRT (β for CRAE: 0.001 (95%CI −0.003, 0.006); p = 0.562 and β for CRVE: 0.002 (95%CI −0.005, 0.010); p = 0.520, respectively). Moreover, no associations were found between change in CRAE or CRVE following pulmonary rehabilitation and change in six-minute walk distance (β for CRAE: −0.004 (95%CI −0.030, 0.021); p = 0.754 and β for CRVE: 0.003 (95%CI −0.036, 0.043); p = 0.868, respectively) and cycle endurance time on CWRT (β for CRAE: −0.004 (95%CI −0.007, 0.003); p = 0.517 and β for CRVE: −0.001 (95%CI −0.006, 0.004); p = 0.768, respectively).

## Discussion

This is the first study measuring retinal vessel diameters in a large group of well-characterized COPD patients. We showed that retinal arteriolar and venular diameters were independently associated with systolic blood pressure, whereas retinal venular diameter was also related to systemic inflammatory status and age. Retinal vessel diameters, as a proxy for microvascular health, did not change after an exercise-based pulmonary rehabilitation program. Retinal vessel diameters were not associated with important functional outcomes of pulmonary rehabilitation. Additionally, we found retinal abnormalities in more than half of the patients with COPD.

To date, only few studies examined the relation between retinal vascular parameters and lung function^26,27,30^. Data from one large population-based study demonstrated that changes in retinal venular diameter were associated with decrements in lung function in healthy individuals (28 mL decrement in FEV_1_ per SD unit of CRVE), especially in current smokers (93 mL decrement in FEV_1_ per SD unit of CRVE)^26^. This association between retinal vessel diameter and lung function was not confirmed by our data from a large group of COPD patients. This might be explained by the compromised lung function with a narrower range of FEV_1_ values in our population compared to the study of Harris *et al*.^26^. In addition, our FEV_1_ values are at the lower bound of the distribution of values measured in the latter study. Similar to our findings, Chew *et al*. and Ugurlu *et al*. did not demonstrate an association between retinal vessel diameter and severity of COPD^27,30^.

Structural assessment of retinal microvasculature can provide useful information for cardiovascular risk prediction^16–18^. To date, no studies have evaluated retinal vessel parameters and cardiovascular risk parameters in COPD. Although earlier population-based studies demonstrated that a lower FEV1 is an independent risk factor for cardiovascular morbidity and mortality^31,32^, we could not establish an association between lung function parameters and retinal vessel diameter in patients with COPD. Then again, we did find an association between retinal vessel diameter and traditional cardiovascular risk factors, including systolic blood pressure and level of inflammation. Considering the high prevalence and incidence of cardiovascular comorbidity in COPD patients^2,33^, and the fact that cardiovascular comorbidities are often undiagnosed^34,35^, an easy to obtain fundus image may be an interesting metric for further cardiovascular phenotyping of patients with COPD. However, we emphasize the need for determining the clinical relevance of retinal imaging in cardiovascular risk prediction in patients with COPD.

It is increasingly recognized that systemic inflammatory markers are involved in the pathogenesis of cardiovascular diseases and can predict cardiovascular events^36,37^, and systemic inflammatory processes may have a role in the association between retinal vessel diameter and cardiovascular diseases, including coronary heart disease, DM, hypertension and stroke^38^. Large population-based studies already demonstrated that systemic inflammation is associated with retinal venular diameter. The Rotterdam study, the multi-ethnic study of atherosclerosis and the Beaver Dam Eye Study reported associations of nonspecific and specific systemic inflammatory markers (i.e. white blood cell count, erythrocyte sedimentation rate, high-sensitive C-reactive protein (hs-CRP), interleukin (IL)−6) with larger venular diameter^18,38,39^. Our study is the first to establish an association between systemic inflammation and retinal venular diameter in patients with COPD. Moreover, this association was consistently seen regardless of smoking status, and other cardiovascular risk factors. In addition, we identified an independent association between retinal vessel diameter and systolic blood pressure. The inverse relation between retinal vessel diameter and elevated systolic blood pressure has already been reported in several large population-based studies^18,21,40^, but, to the best of our knowledge, never in patients with COPD. Future longitudinal studies will be needed to demonstrate the usefulness of retinal blood vessel analysis as part of cardiovascular risk screening in patients with COPD.

In contrast to population-based studies, our findings did not demonstrate an association between retinal venular diameter and blood glucose level or DM. To date, little is known on the exact mechanisms for the association of wider retinal venular diameter with hyperglycemia and DM,. It has been speculated that venular widening may reflect systemic inflammatory processes involved in the pathogenesis of impaired glucose metabolism^41^, which is further supported by findings from epidemiological studies, showing an association between wider retinal venules and elevated systemic inflammatory markers^18,38,39^. It is therefore possible that the high proportion of patients with increased levels of systemic inflammation in this study might attenuate the association between retinal venular diameter and DM. This is confirmed by the study of Heitmar *et al*., in which no association was found between retinal vessel diameter and DM in different patients groups with increased levels of inflammation^42^.

It has been suggested that hypoxia may be involved in the etiology of some retinal abnormalities in COPD^43,44^. Indeed, a constant oxygen supply is crucial for adequate organ function^44^., andeven small changes in oxygen supply to the retina can result in tissue hypoxia and retinal changes^44^. In addition, exposure to corticosteroids may contribute to an increased risk of glaucoma and cataract, though results in patients with COPD are contradictory^45–47^. An earlier review demonstrated that structural and functional changes in the retinal microvasculature are more common and severe in patients with COPD compared to non-COPD controls, including increased retinal vessel diameter, lower retinal arterial oxygen saturation, impaired haemodynamics and increased resistive index of the orbital vessels^43^. Chew *et al*. also demonstrated that retinal abnormalities were more frequently present in hospital patients with COPD compared to other hospital patients (80% vs. 50%)^27^. Furthermore, patients with COPD were more likely to report visual impairment compared to subjects without COPD (14.0% vs. 9.6%)^48^. In line with this, ophthalmological screening in this study identified a high prevalence of retinal abnormalities in patients with COPD. More interestingly, these abnormalities were not recorded in their medical history. As retinal abnormalities can result in a reduced visual acuity^49^, which is related to a decreased quality of life and physical functioning, and increased risk of falling^50^, an ophthalmological screening might be valuable in patients with COPD.

Lower levels of physical activity are associated with wider retinal venular diameters^51,52^. In addition, it has been suggested that physical exercise has a positive effect on the structure (i.e. retinal arteriolar dilatation and venular constriction) and functionality of the microvasculature in subjects with microvascular diseases and physically inactive adults^28,53,54^. To date, only few studies evaluated the effects of different exercise modalities on microvasculature^29,55,56^. In patients with migraine and patients with unipolar depression it has been demonstrated that high-intensity interval training is slightly more effective in improving microvascular structure and function than moderate, continuous exercise^55,56^. It remains challenging to determine which exercise modalities are the most optimal for improving microvascular structure and function for different patient groups and disease severities. Indeed, this is the first study in patients with COPD. Our results demonstrate that an exercise-based pulmonary rehabilitation program did not affect static measurements of retinal vessel diameter in patients with COPD. Although exercise training (i.e. high-intensity interval and continuous) and duration of the pulmonary rehabilitation program were comparable, or even longer compared to earlier studies, it is possible that this program may not provide optimal stimuli to induce significant effects in retinal vessel diameters^56^. Moreover, the timing of pulmonary rehabilitation may not be optimal to improve microvascular function. Indeed, it has been recognized that microvascular dysfunction is an early event in the natural history of COPD^57,58^, and it is an open issue whether microvascular dysfunction is still reversible in patients with advanced COPD. It is possible that the response to an exercise program can be attenuated in patients with COPD, as endothelial dysfunction and impaired flow-mediated dilation are often present in these patients^59^. Moreover, the possible effects of exercise training might be influenced by the high prevalence of cardiovascular comorbidities and use of cardiovascular medication. Similar to our findings, it was shown that pulmonary rehabilitation does not appear to be effective in improving arterial stiffness and endothelial dysfunction in patients with COPD^60,61^. Future studies should investigate the most suitable timing and exercise regimes for improving microvascular structure and function. In this context, one should also consider a more integral assessment of the microvasculature and include the analysis of biomarkers of endothelial function (e.g. endothelin) and functional markers such as dynamic retinal imaging and Laser-Doppler flowmetry^62^.

The major strength of this study is the use of a wide array of clinical variables, cardiovascular risk factors, and functional outcomes, allowing a comprehensive analysis of possible variables related to retinal vessel diameter in a large group of well-characterized COPD patients. This study has the following limitations. First, this is an observational study and no causal relations can be established. Furthermore, it is possible that cardiovascular diseases preceded the development of COPD in some patients. Future studies are needed to determine the clinical relevance of retinal vessel parameters for the development of cardiovascular diseases in patients with COPD. Second, we did not include a healthy control group. Then again, it was already documented that patients with COPD have increased retinal arteriolar and venular diameters compared to non-COPD controls^27,30^. Furthermore, 20% of the patients in this study had retinal vessel calibers outside the earlier reported age- and sex-specific reference range (male: CRAE 129.15–202.49 µm; CRVE 170.52–241.99 µm and female: CRAE 145.92–217.07 µm; CRVE 178.21–259.72 µm)^63^. However, comparison with normative data should be interpreted with caution, as studies used different retinal vessel measurement software systems. It has been demonstrated that there is a poor agreement between different retinal measurement software, indicating that absolute measurements from the different software systems could not be interpreted interchangeably^64,65^. Third, static retinal vessel imaging only reflects a snapshot of the retinal microvasculature and provides limited information on functional alterations of the vasculature^66,67^. For future studies, we recognize the added-value of dynamic vessel analysis because this test has the potential to assess microvascular reactivity and autoregulation, which are suggested to have diagnostic potential in detecting subclinical endothelial dysfunction in the microvasculature and facilitate early diagnosis of cardiovascular diseases^66,67^. Fourth, we only quantified retinal vessel diameter, whilst other retinal vessel parameters, including vessel tortuosity and fractal dimension, may also provide valuable information regarding cardiovascular risk^68^. Fifth, proportion of patient in GOLD stage I and GOLD A were relatively small, and therefore caution is required when generalizing findings to the whole COPD population. Sixth, we only used hs-CRP as marker of systemic inflammation, and we did not measure other inflammatory markers that may be of interest in COPD, such as IL-6, tumor necrosis factor- α, and fibrinogen. Finally, a high proportion of patients had cardiovascular comorbidities, which may have influenced the findings of this study. However, multivariate analyses did not show an association between retinal vessel diameter and cardiovascular diseases or cardiovascular medication. Furthermore, additional analyses did not show significant differences in retinal vessel diameters between patients with and without cardiovascular diseases.

To conclude, we demonstrated that retinal arteriolar and venular diameters of patients with COPD were significantly associated with systolic blood pressure and level of systemic inflammation, but there was no evidence for an association with lung function parameters, functional outcomes or other cardiovascular risk factors. Furthermore, an exercise-based pulmonary rehabilitation program did not affect retinal vessel diameter. Future studies are needed to evaluate the association between physical activity level and retinal vessel diameters and the possible beneficial effect of increased physical activity level on microvascular health, as assessed by retinal vessel diameters, in patients with COPD.

## Methods

### Study design

This was a prospective observational study conducted in Ciro (Horn, the Netherlands)^69^. The study was approved by the Medical Research Ethics Committees United (MEC-U, Nieuwegein, the Netherlands; NL56813.100.16). The study was performed in accordance with the tenets of the Declaration of Helsinki and Good Clinical Practice and has been registered on www.trialregister.nl (NTR5896) before enrolment of the first volunteering participant. All subjects gave written informed consent.

### Study population

Patients referred for clinical assessment and pulmonary rehabilitation to Ciro were screened^69^. Patients were eligible to participate when they were diagnosed with COPD and were clinically stable. Patients with a clinical diagnosis of abnormalities in the lens or cornea, which makes it impossible to image the retina with a fundus camera, were excluded. Additionally, patients with known retinal diseases were excluded. Furthermore, patients who were unable to provide informed consent due to cognitive problems or unable to speak and understand Dutch or English were excluded.

### Exercise-based pulmonary rehabilitation program

All patients with COPD participated in a comprehensive pulmonary rehabilitation program at Ciro, as defined by the latest international ATS/ERS statement on pulmonary rehabilitation^70^. The program consists of 40 sessions and can be inpatient (8 weeks, 5 days/week) or outpatient (8 weeks, 3 half days/week, followed by 8 weeks 2 half days/week). In addition to progressive physical exercises, such as high-intensity interval and continuous training on a stationary cycle and treadmill, and strengthening exercises, the program also included non-exercising components such as occupational therapy, nutritional counselling, psychosocial counselling, exacerbation management, optimizing medication use, and/or educational sessions.

### Measurements

Demographics and clinical characteristics, including BMI, FFMI, post-bronchodilator pulmonary function (FEV_1_, FVC, TLC, RV, and TL_CO_ %predicted), SaO_2_, arterial partial pressure of oxygen (PaO_2_) and carbon dioxide (PaCO_2_), peak aerobic capacity (CPET), functional exercise capacity (6MWT and CWRT at 75% of peak work rate), blood pressure, and blood measurements (i.e. blood glucose, blood lipids (high-density lipoprotein (HDL), LDL, triglycerides), and hs-CRP), were obtained during routine assessment prior and after pulmonary rehabilitation, as described before^71^. Diagnosis of hypertension was based on the use of antihypertensive medication or a systolic blood pressure>140 mmHg and/or diastolic blood pressure>90 mmHg on three separate occasions^72^. The diagnosis of (pre-) DM was based on a fasting plasma glucose level ≥6.1 mmol/L. Dyslipidemia was defined as total cholesterol  ≥6.4 mmol/L, LDL cholesterol  ≥4.4 mmol/L, HDL cholesterol <0.9 mmol/L, triglycerides ≥1.94 mmol/L, or if receiving lipid-lowering medication. Hs-CRP was used as a marker of systemic inflammation and categorized using the established clinical cutoff points into low (<1 mg/L), borderline (1–3 mg/L), moderately high (3.01–10 mg/L) and markedly high (>10 mg/L)^73^. Medication use and cardiovascular comorbidities were obtained from patients’ medical records.

### Retinal photography

A non-mydriatic Canon CR-2 fundus camera (Hospithera, Belgium) with a field of view of 45° was used to obtain high-resolution, optic disc centred images of the fundus of the right eye of each patient at start and end of pulmonary rehabilitation. Patients had to abstain from exercise in the hour prior to the measurement. A trained grader masked to patient characteristics, analyzed the retinal images using semi-automated vessel analysis software developed at VITO (Belgium; http:\\mona.health).

The six largest arterioles and venules coursing through a zone between 0.5 and 1 disc diameter from the optic disc margin were measured and summarized as CRAE and CRVE, representing the average diameter of arterioles and venules of the eye^15,74^. Images were not synchronized on the cardiac cycle, but instead retinal vessel diameters of two images were averaged to minimize random variation in retinal vessel diameter due to different stages of the cardiac cycle^75^.

In addition, all images were screened by two ophthalmologist for the following changes: retinal vascular changes (i.e. increased vessel tortuosity, increased venous diameter, retinal hemorrhage, pathological arterio-venous crossings), optic disc changes (i.e. glaucomatous changes/suspect of glaucoma, peripapillary atrophy, peripapillary bleeding, congenital anatomical optic nerve variation, tilted disc, pale optic disc), vitreoretinal or choroidal structural changes (i.e. macular glistening/epiretinal membrane, foveal/perifoveal pigmentary changes, hard exudates, drusen, choroidal nevi). Slightly blurry media was defined as (suspicious of) cataract.

### Statistics

*A priori*, we have calculated the minimum sample size to achieve adequate power. Based on Chew *et al*.^27^, demonstrating that microvascular abnormalities were prevalent in 80% of the patients with COPD, we should include 246 participants (relative precision of 10% with a 95% confidence interval).

Data were tested for normality and are presented as mean and standard deviation (SD) unless noted otherwise. Age- and sex-adjusted stepwise multiple regression models were used to identify the association between retinal vessel diameter and lung function parameters, clinical variables, cardiovascular risk factors and functional outcomes of pulmonary rehabilitation (6MWT, cycle endurance time on CWRT). Variables were selected as possible confounders if they showed a significant association with retinal vascular width on univariate analysis (p ≤ 0.10), or if a variable was reported to be associated with retinal vessel width in previous literature. In case of high collinearity between two variables, the variable with greater clinical significance, based on the judgement of the authors, was chosen. Variables were included in the final model (Model 1) if they were significant (p ≤ 0.05) and/or they modified the estimates of the remaining parameters (>10% change). To account for potential confounding, models were additionally adjusted for fellow vessel diameter (i.e. for CRVE in models of CRAE, and vice versa^76^) (Model2). Benjamini-Hochberg corrections were applied to correct for multiple testing. General linear models for repeated measures were used to determine the effect of pulmonary rehabilitation on retinal vessel diameter for all patients with COPD and after stratification for response on pulmonary rehabilitation based on the minimal clinically important difference of the 6MWT and cycle endurance time (responder: +30 m on 6MWT^77^ and/or +100 s in cycle endurance time on CWRT^78^, non-responder: <30 m on 6MWT and <100 s in cycle endurance time on CWRT). All analyses were performed using SPSS 25.0 (SPSS Inc; Chicago, Illinois). Level of significance was set at ≤0.05.

## Supplementary information

Supplementary Tables.

## Data Availability

The datasets generated during and/or analysed during the current study are available from the corresponding author on reasonable request

## References

[1] The Global Initiative for Chronic Obstructive Lung Disease (GOLD). Global Strategy for the *Diagnosis, Management and Prevention of Chronic Obstructive Pulmonary Disease*: Report, http://www.goldcopd.org (2019).

[2] Vanfleteren LE (2013). Clusters of comorbidities based on validated objective measurements and systemic inflammation in patients with chronic obstructive pulmonary disease. Am. J. Respir. Crit. Care Med..

[3] Budweiser S, Harlacher M, Pfeifer M, Jorres RA (2014). Co-morbidities and hyperinflation are independent risk factors of all-cause mortality in very severe COPD. COPD.

[4] Antonelli Incalzi R (1997). Co-morbidity contributes to predict mortality of patients with chronic obstructive pulmonary disease. Eur. Respir. J..

[5] Chen W, Thomas J, Sadatsafavi M, FitzGerald JM (2015). Risk of cardiovascular comorbidity in patients with chronic obstructive pulmonary disease: a systematic review and meta-analysis. Lancet Respir. Med..

[6] Vestbo J (2013). Global strategy for the diagnosis, management, and prevention of chronic obstructive pulmonary disease: GOLD executive summary. Am. J. Respiratory Crit. Care Med..

[7] Gibson, G., Loddenkemper, R., Sibille, Y. & Lundbäck, B. The European Lung White Book. (European Respiratory Society, 2013). 10.1183/09031936.00105513 24000245

[8] Leone A, Landini L (2013). Vascular pathology from smoking: look at the microcirculation!. Curr. Vasc. Pharmacology.

[9] Tuder RM, Petrache I (2012). Pathogenesis of chronic obstructive pulmonary disease. J. Clin. Investigation.

[10] Maclay JD, MacNee W (2013). Cardiovascular disease in COPD: mechanisms. Chest.

[11] Macnee W, Maclay J, McAllister D (2008). Cardiovascular injury and repair in chronic obstructive pulmonary disease. Proc. Am. Thorac. Soc..

[12] McClintic BR, McClintic JI, Bisognano JD, Block RC (2010). The relationship between retinal microvascular abnormalities and coronary heart disease: a review. Am. J. Med..

[13] Mimoun L, Massin P, Steg G (2009). Retinal microvascularisation abnormalities and cardiovascular risk. Arch. Cardiovasc. Dis..

[14] Flammer AJ (2012). The assessment of endothelial function: from research into clinical practice. Circulation.

[15] De Boever, P., Louwies, T., Provost, E., Int Panis, L. & Nawrot, T. S. Fundus photography as a convenient tool to study microvascular responses to cardiovascular disease risk factors in epidemiological studies. *J Vis Exp*, e51904, 10.3791/51904 (2014). 10.3791/51904 PMC4353376 25407823

[16] Wong TY (2002). Retinal arteriolar narrowing and risk of coronary heart disease in men and women. The Atherosclerosis Risk in Communities Study. JAMA.

[17] Wong TY (2001). Retinal microvascular abnormalities and incident stroke: the Atherosclerosis Risk in Communities Study. Lancet.

[18] Wong TY (2006). Retinal v0000-0002-4136-1553ascular caliber, cardiovascular risk factors, and inflammation: the multi-ethnic study of atherosclerosis (MESA). Invest. Ophthalmol. Vis. Sci..

[19] Wang JJ (2007). Retinal vessel diameter and cardiovascular mortality: pooled data analysis from two older populations. Eur. Heart J..

[20] Ikram MK, Ong YT, Cheung CY, Wong TY (2013). Retinal vascular caliber measurements: clinical significance, current knowledge and future perspectives. Ophthalmologica.

[21] Smith W (2004). Retinal arteriolar narrowing is associated with 5-year incident severe hypertension: the Blue Mountains Eye Study. Hypertension.

[22] Klein R, Klein BE, Moss SE, Wong TY (2007). Retinal vessel caliber and microvascular and macrovascular disease in type 2 diabetes: XXI: the Wisconsin Epidemiologic Study of Diabetic Retinopathy. Ophthalmology.

[23] Guo VY (2016). Retinal Information is Independently Associated with Cardiovascular Disease in Patients with Type 2 diabetes. Sci. Rep..

[24] Nagele MP (2018). Retinal microvascular dysfunction in heart failure. Eur. Heart J..

[25] Anyfanti, P. et al. Retinal vessel morphology in rheumatoid arthritis: Association with systemic inflammation, subclinical atherosclerosis, and cardiovascular risk. *Microcirculation* 24, 10.1111/micc.12417 (2017). 10.1111/micc.12417 28926162

[26] Harris B (2012). The association of systemic microvascular changes with lung function and lung density: a cross-sectional study. PLoS One.

[27] Chew SK (2016). Hypertensive/Microvascular Disease and COPD: a Case Control Study. Kidney Blood Press. Res..

[28] Hanssen H (2011). Exercise-induced alterations of retinal vessel diameters and cardiovascular risk reduction in obesity. Atherosclerosis.

[29] Louwies T (2019). Microvascular reactivity in rehabilitating cardiac patients based on measurements of retinal blood vessel diameters. Microvasc. Res..

[30] Ugurlu, E. et al. New aspect for systemic effects of COPD: eye findings. *Clin Respir J*, 10.1111/crj.12523 (2016). 10.1111/crj.12523 27401776

[31] Sin DD, Wu L, Man SF (2005). The relationship between reduced lung function and cardiovascular mortality: a population-based study and a systematic review of the literature. Chest.

[32] Ching SM (2019). FEV1 and total Cardiovascular mortality and morbidity over an 18 years follow-up Population-Based Prospective EPIC-NORFOLK Study. BMC Public. Health.

[33] Mullerova H, Agusti A, Erqou S, Mapel DW (2013). Cardiovascular comorbidity in COPD: systematic literature review. Chest.

[34] Rutten FH (2005). Unrecognized heart failure in elderly patients with stable chronic obstructive pulmonary disease. Eur. Heart J..

[35] Houben-Wilke S (2017). Echocardiographic abnormalities and their impact on health status in patients with COPD referred for pulmonary rehabilitation. Respirology.

[36] Willerson JT, Ridker PM (2004). Inflammation as a cardiovascular risk factor. Circulation.

[37] Libby P (2002). Inflammation in atherosclerosis. Nature.

[38] Klein R, Klein BE, Knudtson MD, Wong TY, Tsai MY (2006). Are inflammatory factors related to retinal vessel caliber? The Beaver Dam Eye Study. Arch. Ophthalmol..

[39] Ikram MK (2004). Are retinal arteriolar or venular diameters associated with markers for cardiovascular disorders? The Rotterdam Study. Invest. Ophthalmol. Vis. Sci..

[40] Ikram MK (2006). Retinal vessel diameters and risk of hypertension: the Rotterdam Study. Hypertension.

[41] Nguyen TT, Wang JJ, Wong TY (2007). Retinal vascular changes in pre-diabetes and prehypertension: new findings and their research and clinical implications. Diabetes Care.

[42] Heitmar R, Lip GYH, Ryder RE, Blann AD (2017). Retinal vessel diameters and reactivity in diabetes mellitus and/or cardiovascular disease. Cardiovasc. Diabetol..

[43] Vaes AW (2018). Looking into the eye of patients with chronic obstructive pulmonary disease: an opportunity for better microvascular profiling of these complex patients. Acta Ophthalmol..

[44] Milkowska-Dymanowska J, Bialas AJ, Zalewska-Janowska A, Gorski P, Piotrowski WJ (2015). Underrecognized comorbidities of chronic obstructive pulmonary disease. Int. J. Chron. Obstruct Pulmon Dis..

[45] Nath T (2017). Prevalence of Steroid-Induced Cataract and Glaucoma in Chronic Obstructive Pulmonary Disease Patients Attending a Tertiary Care Center in India. Asia Pac. J. Ophthalmol. (Phila.).

[46] Miller DP, Watkins SE, Sampson T, Davis KJ (2011). Long-term use of fluticasone propionate/salmeterol fixed-dose combination and incidence of cataracts and glaucoma among chronic obstructive pulmonary disease patients in the UK General Practice Research Database. Int. J. Chron. Obstruct Pulmon Dis..

[47] Gartlehner G, Hansen RA, Carson SS, Lohr KN (2006). Efficacy and safety of inhaled corticosteroids in patients with COPD: a systematic review and meta-analysis of health outcomes. Ann. Fam. Med..

[48] Schnell K (2012). The prevalence of clinically-relevant comorbid conditions in patients with physician-diagnosed COPD: a cross-sectional study using data from NHANES 1999-2008. BMC Pulm. Med..

[49] Flaxman SR (2017). Global causes of blindness and distance vision impairment 1990-2020: a systematic review and meta-analysis. Lancet Glob. Health.

[50] Chou R, Dana T, Bougatsos C, Grusing S, Blazina I (2016). Screening for Impaired Visual Acuity in Older Adults: Updated Evidence Report and Systematic Review for the US Preventive Services Task Force. Jama.

[51] Tikellis G, Anuradha S, Klein R, Wong TY (2010). Association between physical activity and retinal microvascular signs: the Atherosclerosis Risk in Communities (ARIC) Study. Microcirculation.

[52] Anuradha S (2011). Physical activity, television viewing time, and retinal microvascular caliber: the multi-ethnic study of atherosclerosis. Am. J. Epidemiol..

[53] Hurley DM (2018). Aerobic Exercise Improves Microvascular Function in Older Adults. Med. Sci. Sports Exerc..

[54] Lanting SM, Johnson NA, Baker MK, Caterson ID, Chuter VH (2017). The effect of exercise training on cutaneous microvascular reactivity: A systematic review and meta-analysis. J. Sci. Med. Sport..

[55] Hanssen H (2018). Effects of different endurance exercise modalities on migraine days and cerebrovascular health in episodic migraineurs: A randomized controlled trial. Scand. J. Med. Sci. Sports.

[56] Hanssen H (2018). Effects of different endurance exercise modalities on retinal vessel diameters in unipolar depression. Microvasc. Res..

[57] Oelsner EC (2019). Albuminuria, Lung Function Decline, and Risk of Incident Chronic Obstructive Pulmonary Disease. The NHLBI Pooled Cohorts Study. Am. J. Respir. Crit. Care Med..

[58] Wouters EFM, Franssen FM (2019). Chronic Obstructive Pulmonary Disease: Shifting the Paradigm to the Vasculature. Am. J. Respir. Crit. Care Med..

[59] Vaes AW (2017). Endothelial function in patients with chronic obstructive pulmonary disease: a systematic review of studies using flow mediated dilatation. Expert. Rev. Respir. Med..

[60] Vanfleteren LE (2014). Arterial stiffness in patients with COPD: the role of systemic inflammation and the effects of pulmonary rehabilitation. Eur. Respir. J..

[61] Gelinas, J. C. *et al*. Aerobic exercise training does not alter vascular structure and function in chronic obstructive pulmonary disease. *Exp Physiol*, 10.1113/ep086379 (2017). 10.1113/EP086379 28857336

[62] Ikram MK (2013). Retinal vascular caliber as a biomarker for diabetes microvascular complications. Diabetes Care.

[63] Ponto KA (2017). Retinal vessel metrics: normative data and their use in systemic hypertension: results from the Gutenberg Health Study. J. Hypertens..

[64] Yip W (2016). Comparison of Common Retinal Vessel Caliber Measurement Software and a Conversion Algorithm. Transl. Vis. Sci. Technol..

[65] McGrory S (2018). Towards Standardization of Quantitative Retinal Vascular Parameters: Comparison of SIVA and VAMPIRE Measurements in the Lothian Birth Cohort 1936. Transl. Vis. Sci. Technol..

[66] Heitmar R, Summers RJ (2012). Assessing vascular function using dynamic retinal diameter measurements: a new insight on the endothelium. Thromb. Haemost..

[67] Lim M (2013). Systemic associations of dynamic retinal vessel analysis: a review of current literature. Microcirculation.

[68] Liew G, Wang JJ, Mitchell P, Wong TY (2008). Retinal vascular imaging: a new tool in microvascular disease research. Circ. Cardiovasc. Imaging.

[69] Spruit MA, Vanderhoven-Augustin I, Janssen PP, Wouters EF (2008). Integration of pulmonary rehabilitation in COPD. Lancet.

[70] Spruit MA (2013). An official american thoracic society/european respiratory society statement: key concepts and advances in pulmonary rehabilitation. Am. J. Respir. Crit. Care Med..

[71] Spruit MA (2007). Extra-pulmonary features in COPD patients entering rehabilitation after stratification for MRC dyspnea grade. Respir. Med..

[72] 2013 Practice guidelines for the management of arterial hypertension of the European Society of Hypertension (ESH) and the European Society of Cardiology (ESC): ESH/ESC Task Force for the Management of Arterial Hypertension. *J Hypertens* **31**, 1925–1938, 10.1097/HJH.0b013e328364ca4c (2013). 10.1097/HJH.0b013e328364ca4c 24107724

[73] Pearson TA (2003). Markers of inflammation and cardiovascular disease: application to clinical and public health practice: A statement for healthcare professionals from the Centers for Disease Control and Prevention and the American Heart Association. Circulation.

[74] Knudtson MD (2003). Revised formulas for summarizing retinal vessel diameters. Curr. Eye Res..

[75] Knudtson MD (2004). Variation associated with measurement of retinal vessel diameters at different points in the pulse cycle. Br. J. Ophthalmol..

[76] Liew G (2007). Measurement of retinal vascular caliber: issues and alternatives to using the arteriole to venule ratio. Invest. Ophthalmol. Vis. Sci..

[77] Holland AE (2014). An official European Respiratory Society/American Thoracic Society technical standard: field walking tests in chronic respiratory disease. Eur. Respiratory J..

[78] Laviolette L (2008). Assessing the impact of pulmonary rehabilitation on functional status in COPD. Thorax.

