# Supplementary material for: Structural analysis of retinal blood vessels in patients with COPD during a pulmonary rehabilitation program
Source: Sci Rep. 2020 Jan 8;10:31. doi: 10.1038/s41598-019-56997-5 (PMC6949286; doi:10.1038/s41598-019-56997-5)
Supplement: Supplementary file 1 — Supplementary Tables. [file 41598_2019_56997_MOESM1_ESM.docx]

**Structural analysis of retinal blood vessels in patients with COPD**

**during a pulmonary rehabilitation program**

Anouk W. Vaes^1,2^*, Martijn A. Spruit^1,3,4,5^, Karel van Keer^6^, João Barbosa-Breda^6,7,8^,

Emiel F.M. Wouters^1,3^, Frits M.E. Franssen^1,3^, Jan Theunis^2^, Patrick De Boever^2,9^

^1^Research and Education, Ciro, Horn, Netherlands

^2^Health Unit, Flemish Institute for Technological Research (VITO), Mol, Belgium

^3^Department of Respiratory Medicine, Maastricht University Medical Centre (MUMC+), Maastricht, The Netherlands

^4^NUTRIM School of Nutrition and Translational Research in Metabolism, Maastricht, The Netherlands

^5^REVAL - Rehabilitation Research Center, BIOMED - Biomedical Research Institute, Faculty of Rehabilitation Sciences, Hasselt University, Diepenbeek, Belgium

^6^Research Group Ophthalmology, Department of Neurosciences, KU Leuven, Leuven, Belgium

^7^Ophthalmology Department, Centro Hospitalar Sao Joao, Porto, Portugal

^8^Department of Surgery and Physiology, Faculty of Medicine of the University of Porto, Porto, Portugal

^9^Centre for Environmental Sciences, Hasselt University, Hasselt, Belgium

**Supplementary Table S1** Retinal abnormalities

| **Retinal abnormalities** | **N (%)** |
| --- | --- |
| None | 100 (40.7) |
| Slightly/mildly increased vessel turtuosity | 44 (17.9) |
| Peripapillary atrophy | 42 (17.1) |
| (suspicion of) Cataract | 34 (13.8) |
| Glaucoma suspect disc | 17 (6.9) |
| Glaucomatous optic disc | 5 (2.0) |
| Tilted disc | 6 (2.4) |
| Peripapillary bleeding | 6 (2.4) |
| Foveal/perifoveal pigmentary changes | 5 (2.0) |
| Horizontal discs | 4 (1.6) |
| Pale optic disc | 4 (1.6) |
| Pathological crossings | 4 (1.6) |
| Congenital anatomical optic nerve variation | 3 (1.2) |
| Macular glistening (sign of epiretinal membrane) | 3 (1.2) |
| Venous ingurgitation (not turtuosity but diameter) | 2 (0.8) |
| Choroidal nevi | 2 (0.8) |
| Hard drusen | 1 (0.4) |
| Miliary drusen | 1 (0.4) |

**Supplementary Table S2.** Complete multiple regression model of CRAE

|  | | | **Model 1** | | **Model 1** | |
| --- | --- | --- | --- | --- | --- | --- |
|  |  |  | **β**  **(95% confidence interval)** | **P-value** | **β**  **(95% confidence interval** | **P-value** |
| Sex, male* | | | 3.1764 (-2.547 – 8.900) | 0.227 | 2.343 (-3.220 – 7.907) | 0.227 |
| Age, years* | | | -0.224 (-0.439 – -0.009) | 0.042 | -0.193 (-0.402 – -0.016) | 0.047 |
| Body mass index, kg/m^2^ | | | -0.116 (-0.678 – 0.447) | 0.687 | -0.025 (-0.572 – 0.523) | 0.929 |
| Fat free mass index, kg/m^2^ | | | 0.129 (-1.369 – 1.626) | 0.866 | -0.164 (-1.623 – 1.295) | 0.825 |
| Smoking | | Current  Ex  Never | 5.602 (-2.147 – 13.350)  5.205 (-1.898 – 12.309)  0 | 0.156  0.151  . | 7.284 (-0.287 – 14.845)  6.292 (-0.616 – 13.199)  0 | 0.059  0.074  . |
| Smoking, packyears | | | -0.058 (-0.126 – 0.009) | 0.092 | -0.060 (-0.126 – 0.005) | 0.072 |
| FEV_1_/FVC | | | 0.060 (-0.111 – 0.231) | 0.493 | 0.095 (-0.072 – 0.262) | 0.266 |
| TL_CO_, % pred | | | -1.047 (-2.353 – 0.259) | 0.116 | -1.149 (-2.416 – 0.118) | 0.075 |
| RV, % pred | | | 0.415 (-2.235 – 3.065) | 0.759 | 0.649 (-1.944 – 3.220) | 0.621 |
| TLC, % pred | | | -0.840 (-3.229 – 1.549) | 0.491 | -0.519 (-2.840 – 1.802) | 0.661 |
| Long-term oxygen therapy | | | 3.143 (-1.341 – 7.627) | 0.169 | 3.033 (-1.312 – 7.378) | 0.171 |
| PaO_2_, kPa | | | -0.377 (-1.558 – 0.804) | 0.531 | -0.190 (-1.339 – 0.959) | 0.746 |
| Systolic blood pressure, mmHg | | | -0.136 (-0.210 – -0.062) | <0.001 | -0.120 (-0.193 – -0.048) | <0.001 |
| Diastolic blood pressure, mmHg | | | -0.084 (-0.241 – 0.037) | 0.294 | -0.055 (-0.208 – 0.098) | 0.481 |
| Blood glucose, mmol/L | | | -0.489 (-2.159 – 1.181) | 0.566 | -0.178 (-1.805 – 1.448) | 0.830 |
| Total cholesterol | | | -0.586 (-10.459 – 9.287) | 0.907 | -1.840 (-11.430 – 7.750) | 0.707 |
| HDL | | | -1.877 (-11.801 – 8.046) | 0.711 | -0.802 (-10.435 – 8.830) | 0.870 |
| LDL | | | 0.255 (-9.569 – 10.106) | 0.959 | 1.372 (-8.192 – 10.936) | 0.779 |
| Triglycerides | | | -1.147 (-5.395 – 3.102) | 0.597 | -0.626 (-4.752 – 3.500) | 0.766 |
| hs-CRP | low  borderline  moderately high  markedly high | | 3.459 (-1.886 – 8.804)  2.803 (-2.361 – 7.967)  2.925 (-2.289 – 8.138)  0 | 0.205  0.287  0.272  . | 3.818 (-2.901 – 8.735)  2.876 (-2.611 – 7.142)  2.432 (-2.684 – 8.548)  0 | 0.203  0.209  0.292  . |
| Cardiovascular medication | | | -1.944 (-5.690 – 1.802) | 0.309 | -1.194 (-4.846 – 2.458) | 0.522 |
| CRVE, µm | | | - | - | 0.122 (0.057 – 0.188) | <0.001 |

**Supplementary Table S3.** Complete multiple regression model of CRVE

|  | | | **Model 1** | | **Model 1** | |
| --- | --- | --- | --- | --- | --- | --- |
|  |  |  | **β**  **(95% confidence interval)** | **P-value** | **β**  **(95% confidence interval)** | **P-value** |
| Sex, male* | | | 6.801 (-4.752 – 18.355) | 0.249 | 5.217 (-6.010 – 16.443) | 0.362 |
| Age, years* | | | -0.654 (-1.089– -0.381) | 0.042 | -0.442 (-0.868– -0.083) | 0.044 |
| Body mass index, kg/m^2^ | | | -0.741 (-1.877 – 0.395) | 0.201 | -0.683 (-1.784 – 0.418) | 0.224 |
| Fat free mass index, kg/m^2^ | | | 2.391 (-0.631 – 5.413) | 0.121 | 2.327 (-0.602 – 5.255) | 0.119 |
| Smoking | | Current  Ex  Never | -13.735 (-29.376 – 1.906)  -8.872 (-23.211 – 5.468)  0 | 0.085  0.225  0 | -15.530 (-30.7596 – 0.301)  -11.469 (-25.432 – 2.495)  0 | 0.083  0.107  0 |
| Smoking, packyears | | | 0.016 (-0.121 – 0.152) | 0.823 | 0.045 (-0.089 – 0.178) | 0.511 |
| FEV_1_/FVC | | | -0.284 (-0.630 – 0.061) | 0.107 | -0.314 (-0.649 – 0.021) | 0.066 |
| TL_CO_, % pred | | | 0.837 (-1.799 – 3.474) | 0.534 | 1.360 (-1.210 – 3.930) | 0.300 |
| RV, % pred | | | -1.916 (-7.265 – 3.434) | 0.483 | -2.123 (-7.308 – 3.062) | 0.422 |
| TLC, % pred | | | -2.621 (-7.443 – 2.202) | 0.287 | -2.202 (-6.880 – 2.476) | 0.356 |
| Long-term oxygen therapy | | | 0.897 (-9.947 – 8.154) | 0.846 | 0.672 (-8.138 – 9.482) | 0.881 |
| PaO_2_, kPa | | | -1.528 (-3.912 – 0.857) | 0.209 | -1.340 (-3.652 – 0.973) | 0.256 |
| Systolic blood pressure, mmHg | | | -0.229 (-0.379 – -0.120) | 0.041 | -0.211 (-0.370 – -0.047) | 0.047 |
| Diastolic blood pressure, mmHg | | | -0.238 (-0.555 – 0.079) | 0.141 | -0.196 (-0.504 – 0.112) | 0.213 |
| Blood glucose, mmol/L | | | -2.537 (-5.908 – 0.833) | 0.140 | -2.293 (-5.562 – 0.975) | 0.169 |
| Total cholesterol | | | 10.245 (-9.685 – 30.174) | 0.314 | 10.537 (-8.775 – 29.849) | 0.285 |
| HDL | | | -8.780 (-28.811 – 11.250) | 0.390 | -7.844 (-27.259 – 11.572) | 0.428 |
| LDL | | | -9.117 (-29.002 – 10.768) | 0.369 | -9.244 (-28.513 – 10.024) | 0.347 |
| Triglycerides | | | -4.251 (-12.827 – 4.326) | 0.331 | -3.679 (-11.995 – 4.637) | 0.386 |
| hs-CRP | low  borderline  moderately high  markedly high | | -13.767 (-24.556 – -2.978)  -15.097 (-25.521 – 4.674)  -2.309 (-4.833 – -0.785)  0 | <0.001  <0.001  0.022  . | -15.493 (-25.987 – -4.998)  -16.496 (-26.624 – 6.368)  -3.768 (-6.995 – -0.541)  0 | <0.001  <0.001  0.008  . |
| Cardiovascular medication | | | -6.123 (-13.685 – 1.438) | 0.112 | -5.154 (-12.499 – 2.192) | 0.169 |
| CRAE, µm | | | - | - | 0.499 (0.232 – 0.765) | <0.001 |
